# Beyond the M.D.: Transdisciplinary approaches of high-volume dual degree M.D./Masters programs at U.S. allopathic medical schools

**DOI:** 10.1186/s12909-024-05709-3

**Published:** 2024-07-16

**Authors:** Gauri Agarwal, Brett M. Colbert, Sarah Jacobs, Amar R. Deshpande, Latha Chandran, S. Barry Issenberg

**Affiliations:** Leonard M. Miller School of Medicine, University of Miami, Miami, FL 33136 United States of America

**Keywords:** Dual degree, Joint degree, Transdisciplinary

## Abstract

**Purpose:**

*Transdisciplinarity* has been described as a fusion of theories, methods, and expertise across disciplinary boundaries to address complex, global problems. This approach has coincided with an increase in US medical schools offering masters degrees along with an MD degree to equip medical students to practice in complex, interconnected health systems. This study focused on medical schools that graduate the most dual degree students per year and explored the alignment of such programs with a transdisciplinary approach.

**Methods:**

We identified 19 allopathic medical schools that annually graduated an average of 10 or more dual-degree students from 2015–2020. We surveyed these schools and asked participants to describe the reason(s) their institutions offered dual-degree programs. Two authors coded the narrative responses from the survey.

**Results:**

Responses were received from 17 of the 19 schools. The analysis of participants’ responses regarding their institutions’ purpose for offering dual programs revealed several themes associated with a *transdisciplinar*y approach to training. The most common themes were expand skill sets beyond a medical degree (73%), provide opportunity for interdisciplinary collaboration (67%), expand career interest and goals (60%), develop leaders (53%), enhance residency applications (47%) and further the institution’s vision and mission (45%).

**Conclusions:**

This study is the first comprehensive evaluation of MD/Masters programs in the United States that includes a summary of the medical schools with the largest dual degree programs and their reasons for offering them. The findings support the hypothesis that allopathic medical schools recognize the need for a transdisciplinary approach to prepare students for the complexities in healthcare. These programs provide students with opportunities for additional areas of expertise, leadership development, enhancement of competitiveness for residency application, and interdisciplinary collaboration. Medical schools without dual-degree programs may consider developing these programs to provide benefits to students and institutions.

**Supplementary Information:**

The online version contains supplementary material available at 10.1186/s12909-024-05709-3.

## Practice points

- There has been a growth of medical schools in the United States offering masters programs for medical students.
- Medical schools with the largest number of graduates in these programs feel the additional degree allows for an expansion of skill sets, opportunities for interdisciplinary collaboration, expansion of career goals, leadership development, enhancement of residency application, and support of their institutions’ vision and mission.
- Given the broad areas of benefits to students and institutions described by respondents from our study, medical schools without dual-degree programs should consider exploring the development of these programs.

## Introduction

For more than three decades, it has been recognized that traditional curricula may not be preparing students for a complex, dynamic and increasingly interconnected world. In response to this need, an approach to problem solving has emerged referred to as *transdisciplinarity*, defined as representing “a functional synthesis of methodologies and a broad point of view that combines different fields” [1]. Transdisciplinarity has been described as a fusion of theories, methods and expertise across disciplinary boundaries in which each discipline merges with the others in the formation of a whole that is greater than the sum of its parts. This approach has been suggested to address new, highly complex, global concerns, extending into areas concerning science, technology, social problems and policy, education, and the arts. Transdisciplinarity has been distinguished from multidisciplinary (different disciplines provide their own distinct recommendations) and interdisciplinary (specialists provide recommendations and achieve consensus) because it widens the scope of disciplines that can work together (outside the health professions) and allows these specialists to provide recommendations, achieve consensus, and *share each other’s roles* in order to transcend one’s own area of expertise [14]. As examples, in multidisciplinary rounds, each member of a healthcare team may provide recommendations for the care of a patient but not work together to come up with those recommendations. In interdisciplinary rounds, two disciplines within healthcare may work together to come up with an integrated approach to the care of a patient. However, in a transdisciplinary approach, one discipline learns about and adopts the principles and practice of another discipline (even if outside of healthcare) to come up with a novel solution to a complex problem such as a physician utilizing art or theater to educate a community about a public health principle. This approach has been used in medical schools in various ways such as exploring the connection of human health to ecological and animal systems [14], problem solving to address climate change [18], and working with engineering colleagues to enhance medical innovation [7].

These developments have coincided with an increase in U.S. allopathic medical schools offering Masters degrees to medical students to equip them to practice in more complex, interconnected health systems. Masters programs in the United States are graduate-level programs completed after completion of a bachelor’s degree and are sometimes referred to as “post-graduate” programs in other countries. There have been expanding efforts in medical schools outside of the United States to intercalate degrees such as Master of Health Professions Education [13, 29] and Master of Philosophy [23]. Globally, this movement toward dual/joint degrees is growing and international bodies have recognized the need to develop standards and best practice guidelines [4, 10].

This focus of this study and report is on U.S. allopathic dual MD/Masters programs as there have been several recent publications on the structure and outcomes of these programs. Dual-degree programs provide additional expertise for students outside of clinical training that may help them become better scientists, leaders, and physicians [5, 24, 28]. For example, MD/Master of Business Administration programs (MBA) programs have been developed because physicians increasingly have “combined clinical and administrative functions” and there is a “demand for related training of physician leaders.” [24] A recent literature review of U.S. MD/Master of Public Health (MPH) programs noted that “the additional population health, health care advocacy, and epidemiology skills better prepare physicians to work in complex health systems and communities.” [22]

From the student perspective, value added skills such as creativity, analysis and synthesis, problem definition, and innovation will be required to sustain students as they enter a continually changing work environment. In a survey of MD MBA programs graduates, most respondents indicated that obtaining the MBA led to an acceleration of their career, professional flexibility, and credibility [20]. Dual-degree programs also seem to have an impact on career choice. For example, dual MDMPH training has a stronger effect on producing primary care physicians than participation as an officer in a family medicine interest group [17, 24].

In addition, residency (post-MD) program directors may find added value in recruiting incoming residents with additional expertise in relevant areas, such as health administration, research, or quality improvement. Finally, with the transition of the United States Medical Licensure Exam (USMLE) Step 1 exam to a Pass/Fail system in 2022, a second degree can be an important factor in distinguishing applicants for residency programs. Medical students choose to pursue dual degrees for leadership opportunities, preparation for non-clinical aspects of the practice of medicine, job security, and achievement of personal career goals.

The Association of American Medical Colleges (AAMC) does not publish details regarding the total number of dual-degree graduates in US medical schools, the time required for degree completion, or the size of each dual-degree program. The only publicly available data on dual degree programs is offered through the AAMC website’s curriculum reports which indicate that from 2011–2016, there was a rise in dual degree programs, most notably in MD-Masters programs such as MD/MPH and MD/Master of Science (MS) programs (AAMC) [2]. While prior studies have focused on descriptions and individual institutional and student perspectives of specific dual-degree programs such as MD/MPH and MD/MBA programs [12, 19, 25], there have been no reports that include the institutional perspective of multiple U.S. dual-degree programs (AAMC) [2]. We hypothesized that medical schools offer these programs to provide transdisciplinary training for their students. The purpose of this study was to better understand the phenomenon of dual degree Masters programs in medical schools by choosing to focus on medical schools that graduate the largest number (10 or more per year) of students per year and explore the alignment of such programs with a *transdisciplinary* approach of preparing medical students for a twenty-first century career.

## Methods

### AAMC data request and quantitative analysis

To identify those programs that graduate more than ten students per year, we requested data from the AAMC on all U.S. medical schools’ aggregate matriculant and graduate counts for five academic years from 2015–2016 through 2019–2020 (AAMC) [3]. From the AAMC report, we gathered annual data per school on the total number of dual-degree graduates and the number of dual degrees granted in four years versus more than four years. As the AAMC data did not contain information about which dual-degree programs each institution offered, we reviewed the Medical School Admission Requirements (MSAR) page for each school and added the degrees offered to our data sheet from the “Combined Degrees” section in MSAR. We focused on integrated MD/Masters programs that were completed while completing the MD degree, and not students who entered medical school with a degree prior to matriculation. We also excluded PhD and JD programs as these are lengthy (at least 3 years) and not as easily integrated into MD curricula. We also gathered the per class enrollment from the MSAR “First Year Class” field. The data sheet we compiled was stored in a secure cloud location (Box, Redwood City, CA) and was used for subsequent analyses.

### Participant sampling

**Fig. 1:**
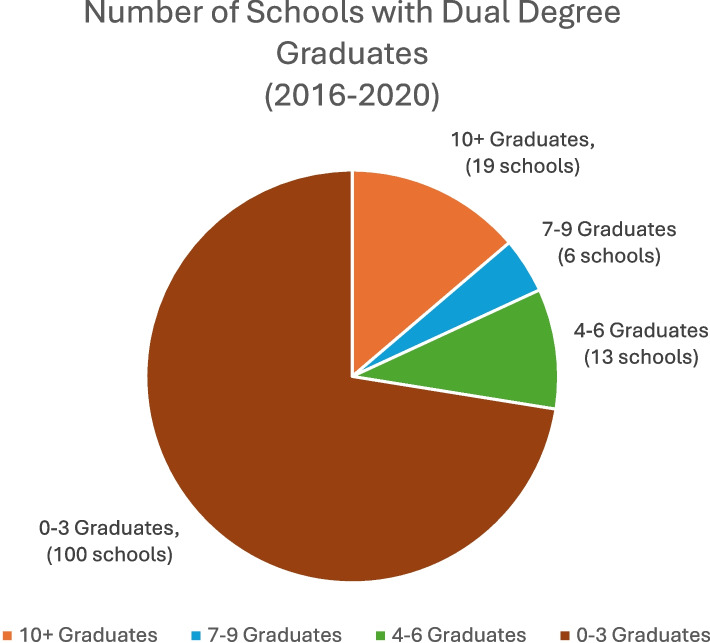
Of the 152 U.S. medical schools, 100 graduate 0–3 dual degree students per year. Thirteen graduate 4–6, six graduate 7–9, and 19 graduate 10 or more. Number of schools was determined by the average number of graduates from 2016–2020

**Fig. 2:**
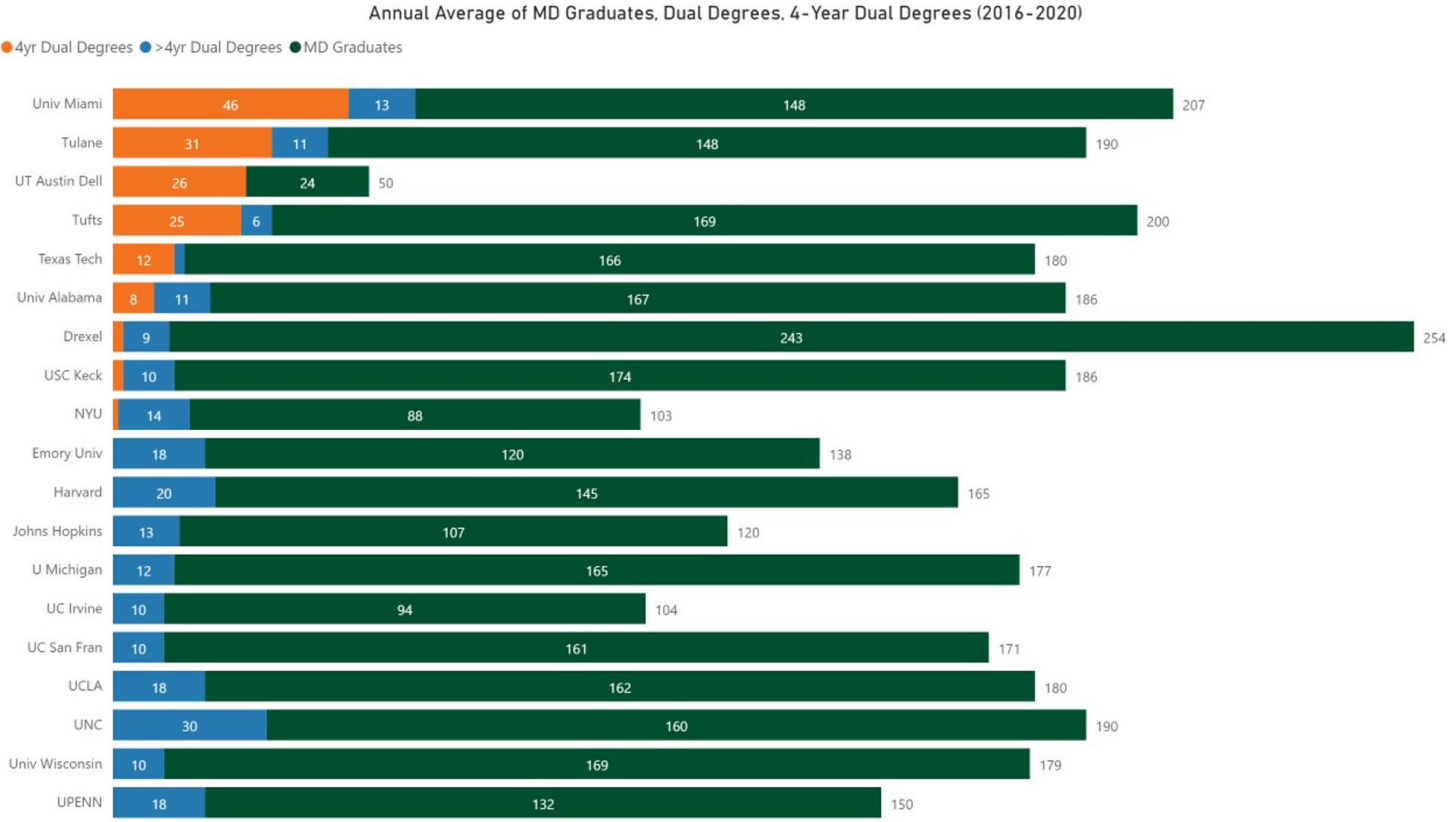
Annual Number of Graduates from U.S. Medical Schools with the Largest number of Dual Degree Graduates. The bar indicates the average number of MD Graduates, 5-Year Dual Degree Graduates, and 4-Year Dual Degree Graduates per school

Of the 152 U.S. medical schools at the time of data collection, 131 (86.2%) offered at least one of the following MD dual-degree programs: Bachelor of Science (BS), MPH, MBA, Master of Science (MS), Master of Arts (MA), Juris Doctor (JD), or Doctor of Philosophy (PhD). Of the 131 medical schools offering dual-degree programs, 65.8% offered MD/Master programs (59.2% MD/MPH, 46.1% MD/MBA, 40.1% MD/MS, 28.3% MD/BS, and 10.5% MD/MA) with 76.9% and 12.5% offering MD/PhD and MD/JD programs respectively. Of the 100 schools with combined MD/Master programs, the number of total graduates per year per school ranged from 1 to 59. As we were interested in schools that graduated a high volume of dual-degree masters students, we focused on the nineteen medical schools that had graduated an average of 10 or more dual degree students per year over the past five years. (Fig. 1) Nineteen of the 100 schools that offered combined MD/Masters programs graduated an average of 10 or more students per year. The average annual number of dual degree graduates for these nineteen schools is shown in Fig. 2. Twenty-six (17.1%) of all 152 U.S. medical schools offered *four-year* MD/Master programs. We excluded medical schools that did not have dual degree graduates for five or more years (except one new medical school that requires all students to choose either a pathway of distinction or dual degree to graduate). We chose a purposive sampling approach as we felt these institutions were likely to have the most breadth of experience for our intended survey and they had demonstrated a consistent institutional commitment over five years to offer these programs [21]. Purposive sampling is a form of non-probability sampling in which researchers rely on their own judgment when choosing members of the population to participate in their surveys. While the findings from purposive sampling are not always statistically representative of the greater population of interest, they are qualitatively generalizable. We felt the sample of the largest dual degree programs we chose would offer perspectives and experiences that would be representative of most dual degree programs.

### Survey development & data collection

We developed a survey using Cognito Forms (Columbia, SC) that included pre-populated data we had acquired from the AAMC as well as several open-ended questions that asked participants to describe the reason(s) their institutions chose to offer dual degree program opportunities for their medical students and details regarding their individual programs. (Appendix 1). In July 2021 we emailed academic deans and directors listed on institutional websites as having oversight of dual degree programs for the nineteen selected schools with a unique link and password to the survey that was open for a 3-month period; email reminders were sent bi-weekly until the conclusion of the survey. Each participant was required to enter an email and unique password to access the survey. Participants were asked to verify the AAMC data regarding each dual-degree program at their institution, make any corrections or updates to the data, and answer the open-ended questions.

The University of Miami Institutional Review Board (IRB number 20210381) approved the study. Informed consent was obtained from all respondents. For the analysis of survey responses, one of the study authors (BC) is a dual-degree student in a MD-PhD program, two of the authors (BI) are medical education deans, and one of the authors (GA) also acted as a contributor to the survey as our institution was identified and included as one of the nineteen schools in the sample.

### Qualitative data analysis

Two members of the study team (B.C. and B.I.) initially coded the narrative responses from the survey using Braun and Clarke’s framework for thematic analysis [6]. Following this methodology, the two coders independently analyzed responses, highlighted material of interest, and annotated them with comments [11]. They generated codes and categorized them into themes based on patterned responses and the richness of responses. An inductive thematic analysis approach was used, which is a process of coding the data without trying to fit them into a pre-existing coding frame or the researchers’ analytic preconceptions. In the inductive approach, the themes identified are strongly linked to the data themselves. In keeping with critical research practice, we employed critical reflexivity to explore our positions on the dataset [16]. We used a series of virtual meetings to discuss our perspectives on the data based on our individual backgrounds; we discussed implicit bias and documented differences in opinions that may have impacted data analysis. An audit trail was maintained, analyzing team meetings and discussing codes, revisions, and team member comments. Member check was done by inviting a co-investigator and survey participant (G.A.) to review themes and affirm that the themes reflected participants’ experiences.

## Results

### Survey analysis of institutional perspectives

We received survey responses from 17 of the 19 schools that met our criteria, with a response rate of 89%. The analysis of the participants’ responses regarding their institutions’ purpose for offering dual programs revealed several themes associated with a *transdisciplinar*y approach to training and a few related to more specific student or institutional reasons. Table 1 details the general themes and dual degree-specific themes with example quotes from the responses.

**Table 1:** Transdisciplinary and Individual Institutional Reasons for Offering Dual Degree Programs in US Medical Schools

TRANSDISCIPLINARY REASONS		
**Main Themes For Institution**	**Investigators Definition**	**Representative Quotes**
**Interdisciplinary collaboration**	Providing opportunity to collaborate with other professions and disciplines	The development of dual degree programs was designed to enhance interdisciplinary collaboration within our community which is a critical part of the mission. The medical school decided to offer dual degree programs because it appreciates and recognizes the importance of multidisciplinary fields coming together to care for patients and the community. Medicine is best practiced with colleagues from multiple specialties. Dual degrees complement each other and help healthcare providers optimally care for patients and populations.
**Recognition of need in medicine** Serve large scale populations Train the future public health professionals Aid the local community	Identified an existing or emerging need that dual degree addresses MD/MPH program enables graduates to apply skills to large population healthcare MD/MPH program goals is to prepare physicians to focus their career in public health MD/MPH program enables institution and students to serve local community	Recognition of emerging areas of need in medicine in general and in our region, in particular, was also a major factor, for example in the establishment of our newest dual degree, an MD/ M.Eng in collaboration with our College of Engineering. In response to the ongoing reorganization of health care delivery systems, and the growing awareness of the impact of business decisions on health care, … we offer an innovative program for students seeking knowledge in both medicine and business administration. The MD/MBA program was established … due to the recognition that medical training did not prepare our graduates appropriately for the challenges inherent in the financial management of health care. The MPH training gives students a "bigger picture" view of the medicine they practice and equips them to affect the outcomes of entire populations rather than solely treating one patient at a time. Training an elite group of physicians in the aspects of public health will create, integrate and translate population-based knowledge into preventive strategies for reducing the societal burden of human disease and disability through excellence in research, education, and public service. Clinically-trained physicians with the MPH degree can take positions in state and local health departments, the United States Centers for Disease Control and Prevention, non-profit organizations such as community health centers, and health plans. They may also work in international contexts. Students in the four-year MD MPH program benefit from a regional campus environment as they are embedded in community practices, health department clinics, community public health immersions, and experience a number of different healthcare delivery models.
**Expand skill set beyond discipline of medicine** Entrepreneurship Prepare students for careers in business Provide Laboratory Experience	Focused development of competencies beyond traditional medical degree MD/MBA program encourages its graduates to be entrepreneurs (in addition to broader business administration skills MD/MBA program focuses on individuals who want to develop expertise in business administration and management in healthcare Provide opportunity to develop laboratory research skills in less time than a MD/PhD	The MPH program gives students additional skills that enable them to better serve the citizens of the state … some areas of which have significant public health challenges. The training … gives students skills … needed for careers in administration, entrepreneurship, economics, finance, consulting, or innovation in healthcare. We recognize the value of the additional organizational leadership education and training, policy analysis, and understanding of public policy institutions in training future physicians to be change agents. We are currently offering a Masters of International Administration in which students learn how to identify, analyze, and manage complex global problems that confront modern multi-national institutions. The program combines academic considerations of geopolitics, culture, organizational leadership, and security studies to provide students with the skills necessary to pursue a range of career objectives in both domestic and international contexts. We have students who have an interest in entrepreneurship and appreciate the ability to collaborate with the many scientists on our campus Our institution “encourages entrepreneurship, and the joint MBA/MD degree prepares these students for the investment and marketing aspects of medicine.” The … program is aimed at individuals … who seek a career as physicians with major responsibility for administration and management in health care organizations and institutions. This program was designed for those students who want a more substantial laboratory research experience while completing their MD degree. To develop one in basic biomedical sciences for students who wish to refine their bench research skills without the extended length of a Ph.D. It is essentially an off-shoot of our MD/PhD program and is designed for students who wish to obtain research experience but are unwilling to commit to the full-time required for the MD/PhD program.
**Create leaders in the field** Prepare students for healthcare leadership of large organizations	The dual degree provides more formal training of leadership skills and opportunities to apply them MD/MBA program prepares students to assume leadership positions in large healthcare organizations	Having additional training makes our graduates more likely to be strong leaders in their eventual fields of practice, and often more equipped to serve their community. The curricular goal is to produce "transformational leaders" in medicine, and we feel these additional skills allow them to do become such leaders. The program … is designed to allow students to rapidly take on roles of responsibility and leadership in the healthcare sector. The … program is designed to prepare its graduates to assume leadership in the design and management of health care systems.
**INDIVIDUAL REASONS**		
** Enhance residency applications**	Providing additional strength for applying to competitive residencies	Our prior dual degree programs have been highly successful with outstanding match outcomes and students who feel they have expanded skill sets that are attractive to residency program directors.
** Attract high quality applicants and faculty**	Offering dual degrees makes the institution more attractive to medical student applicants and faculty	We … recognized that these programs provided a competitive edge in attracting highly talented students to our school We designed to program … to provide it with a competitive advantage and have found that this program is a major factor for a cohort of highly qualified students choosing our school.
** Career interests and goals**	Opportunity for students who are interested in expanding their career interests and goals	Dual/combined degree programs offer students the opportunity to pursue additional, deeper learning in an area that is both an area of their passion and that combines well with the content being learned in medical school in our curriculum.
** Further school mission and vision**	Offering dual degree is aligned with institution’s vision and mission	[–] is a health sciences university that creates knowledge and applies science and discoveries to further education, healthcare and community service locally and globally. The main function of the MD/MPH dual degree program is to support and further that mission We take advantage of our location at the heart of a large university campus with outstanding schools of law, economics and arts & sciences.

### Interdisciplinary collaboration

There were several institutions that offered dual degree programs to provide medical students and faculty opportunities for collaboration with other disciplines.

- *“The development of dual degree programs was designed to enhance interdisciplinary collaboration within our community which is a critical part of the mission.”*
- *“The medical school decided to offer dual degree programs because it appreciates and recognizes the importance of multidisciplinary fields coming together to care for patients and the community. Dual degrees complement each other and help healthcare providers optimally care for patients and populations.”*
- *“Our MD/MA program is an opportunity for students to do graduate level work with individuals focused on the humanities. It was developed by faculty in our … Department of Social Medicine.”*

### Recognition of need in medicine

Several institutions indicated that the development of their dual-degree program was in response to an existing need not previously addressed or an emerging need in medicine.

- *“[We hope to] improve the health of populations, particularly those in underserved communities, and better serve the citizens of [our] state, some areas of which have significant public health challenges.”*
- *“Recognition of emerging areas of need in medicine in general and in our region, in particular, was also a major factor, for example in the establishment of our newest dual degree, an MD/ M. Eng in collaboration with our College of Engineering. In response to the ongoing reorganization of health care delivery systems, and the growing awareness of the impact of business decisions on health care, … we offer an innovative program for students seeking knowledge in both medicine and business administration.”*
- *“The MD/MBA program was established … due to the recognition that medical training did not prepare our graduates appropriately for the challenges inherent in the financial management of health care.”*

### Expand skill set beyond discipline of medicine

Respondents provided many examples about how their dual-degree programs offer medical students the opportunity to develop competencies beyond those typically associated with a medical degree. While the free text narrative for several of the other themes were broad in their descriptions, specific skills (associated with specific programs) were described in this theme:

- *“[MBA] training … gives students skills … needed for careers in administration, entrepreneurship, economics, finance, consulting, or innovation in healthcare.”*
- *“We recognize the value of the additional organizational leadership education and training, policy analysis, and understanding of public policy institutions in [MBA] training [for] future physicians to be change agents.”*
- *“MPH programs give students additional skills that enable them to better serve the citizens of the state … some areas of which have significant public health challenges,” and they are “essential for persons conducting clinical or community-based research, and/or involved with health service organization.”*
- *“We are currently offering a Master of International Administration (MAIA) in which students learn how to identify, analyze, and manage complex global problems that confront modern multi-national institutions…. Students can be prepared for careers that incorporate human rights, disaster management, and security management.”*

### Create leaders in the field

Several respondents commented on the role of dual degree programs in teaching leadership skills and providing opportunities to apply these skills during the training period.

- *“Having additional training makes our graduates more likely to be strong leaders in their eventual fields of practice and often more equipped to serve their community.”*
- *"The [MBA] program confers joint degrees in medicine and business and is designed to allow students to rapidly take on roles of responsibility and leadership in the healthcare sector.”*
- *“Our curricular goal is to produce "transformational leaders" in medicine, and we feel these additional skills allow them to become such leaders.”*

### Expand career interests and goals

Several institutions indicated that their dual degree programs addressed student interests and provided expanded career opportunities.

- *“Dual/combined degree programs offer students the opportunity to pursue additional, deeper learning in an area that is both an area of their passion and that combines well with the content being learned in medical school.”*
- *“Clinically trained physicians with the MPH degree can take positions in state and local health departments, the United States Centers for Disease Control and Prevention, non-profit organizations such as community health centers, and health plans. They may also work in international contexts.”*
- *“We encourage entrepreneurship, and the joint MD/MBA degree prepares these students for the investment and marketing aspects of medicine.”*
- *“Medical students may pursue an additional Master of Science degree in clinical and translational research, we are working to develop one in basic biomedical sciences for students who wish to refine their bench research skills without the extended length of a Ph.D.”*

### Further the institution’s vision and mission

Dual degree programs may also serve a role in enhancing the institution's mission and utilizing its strengths.

- *“[We are] a health sciences university that creates knowledge and applies science and discoveries to further education, healthcare, and community service locally and globally. The main function of the MD/MPH dual degree program is to support and further that mission.”*
- *“We take advantage of our location at the heart of a large university campus with outstanding schools of law, economics, and arts & sciences.”*

### Enhance residency applications

Survey respondents also felt that dual degrees enhanced student applications when applying to residency programs.

- *“Our prior dual degree programs have been highly successful with outstanding match outcomes and students who feel they have expanded skill sets that are attractive to residency program directors.”*
- *“[We hope] to provide additional skills and better equip our graduates to deliver twenty-first century care and improve residency match outcomes”*

### Attract high quality applicants and faculty

Finally, dual degree programs appear to attract unique applicants and faculty to medical schools.

- *“The establishment of a Department of Public Health in the Graduate School of Biomedical Sciences provided us with the opportunity to establish this program, which had been an area of significant interest for faculty (particularly those with MD/MPH degrees themselves) as well as incoming students, who might choose another school simply based on the availability of this type of program.”*
- *“We … recognized that these programs provided a competitive edge in attracting highly talented students to our school.”*
- *“We designed [the MBA] program to be completed in 4 years to provide it with a competitive advantage and have found that this program is a major factor for a cohort of highly qualified students choosing our school.”*

## Discussion

To our knowledge, this study is the first comprehensive evaluation of MD/Masters programs in the United States that includes a summary of the institutions with the largest of these dual degree programs and their reasons for offering them. While a majority of U.S. medical schools offer dual degree opportunities for students, few schools graduate a relatively large (> 10) number of students in these combined MD/Masters programs, and even fewer graduate students within a four-year timeline.

**Fig. 3:**
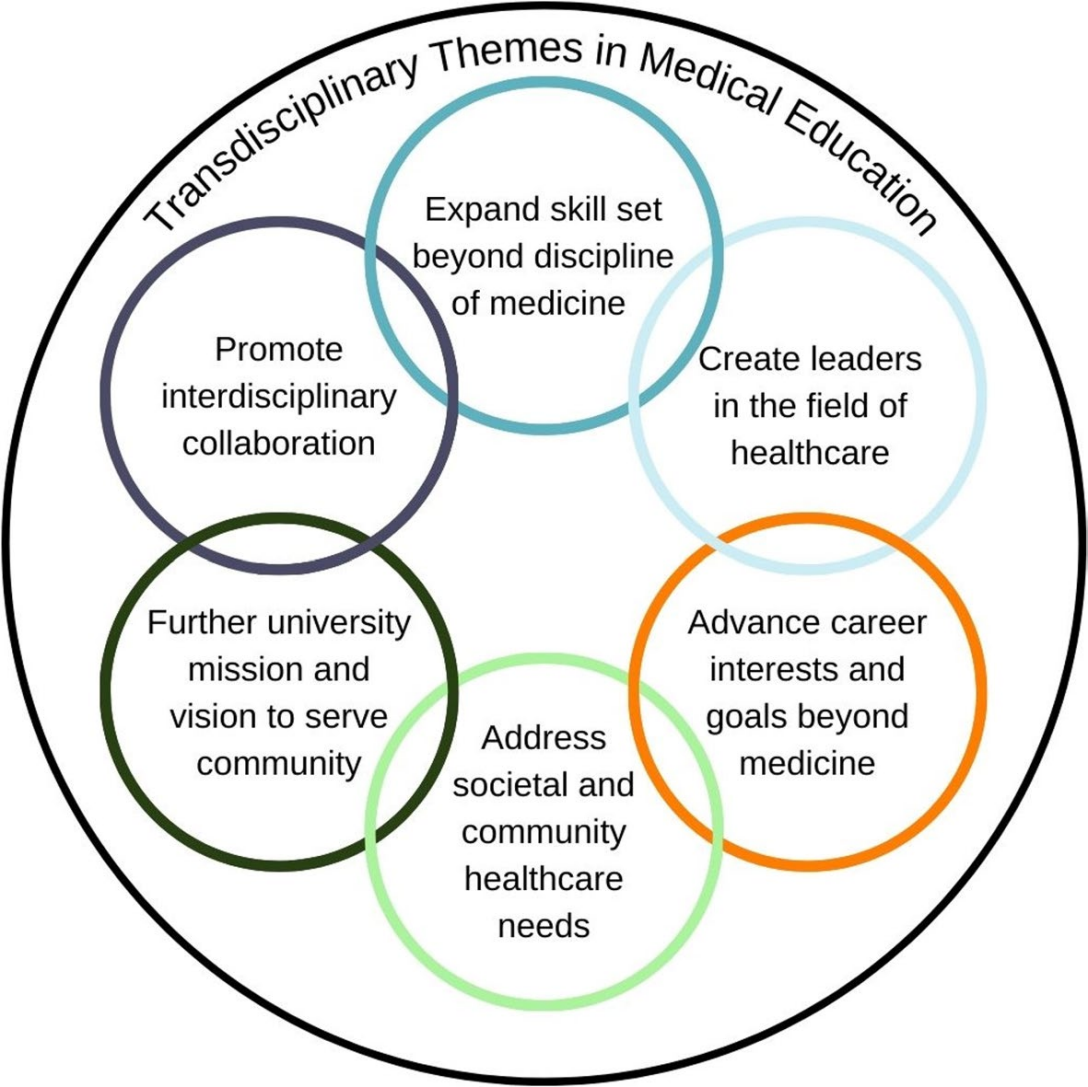
Themes of Transdisciplinarity that Emerged from Allopathic Medical Schools in the United States with many Dual Degree Graduates

Our findings (Fig. 3) revealed several themes consistent with our hypothesis that medical schools are recognizing the need and adopting a transdisciplinary approach to prepare medical students for the growing complexities in healthcare. Dual MD/Masters programs are growing well beyond the more dominant MPH and MBA degrees, with several schools now offering MD/MS programs as alternatives for students interested in research but unable to commit to the extra time and expense of pursuing a PhD [26]. MS programs focus on several areas including genomics, epidemiology, health policy, bioinformatics, and biomedical engineering. These programs provide students with training in applying research methods and opportunities to design and implement research projects. MD/MA programs appear to be wide and varied and often capitalize on institutional strengths such as bioethics, humanities, education, international administration, and integrative well-being. The AAMC recently committed to supporting the integration of arts and humanities in medical education. In 2018, Klugman et al. surveyed 134 U.S. medical schools regarding their humanities content and found that 50.0% of the schools reported only 0–25% of students participating in humanities electives [15]. These programs seem to be addressing gaps not included in a conventional medical education curriculum. These gaps could also be addressed by medical schools without dual-degree programs with enhancements like scholarly pathways of emphasis.

### Transdisciplinarity

Findings from this study revealed that dual degree programs provide greater opportunities for interprofessional work, particularly those related to community-based practice. Crismon et al. recently reviewed the role of dual degree programs in Schools of Pharmacy and commented on the interprofessional aspects noting, *“Given the expansion of dual degree programs over the past decade, … colleges/schools of pharmacy… are signaling to students an acceptance of non-traditional careers either inside or outside the profession…Offering dual degree programs has the potential to stimulate interprofessional and interdisciplinary education, research, and practice, which can also impact the culture of the faculty as well as students.”* [9].

Consistent with prior studies of the benefits of MD/MPH and MD/MBA programs, our analysis reveals that medical schools support these programs to offer students additional areas of expertise and to provide opportunities to become health system and public health leaders [5, 20, 24, 25, 27]. In addition, our study revealed that medical schools offer these programs to meet the needs of their local communities. These needs could encompass serving local underserved populations and applying skills related to knowledge of healthcare administration and business to meet the needs of clinical practice in a region.

### Individual Institutional Reasons

This study’s findings also revealed that leaders feel that dual degree programs can provide students with greater career flexibility, further the school’s mission, attract more competitive applicants and improve residency match outcomes. The latter finding is supported by a recent study by Christensen et al. which utilized voluntary self-reported data from the Texas STAR database from 2017–2019 of 115 medical schools. The study compared residency match outcomes between traditional MD degree students and dual-degree students. There was not a significant difference of obtaining a dual degree on overall match rates with MD/MPH, MD/MBA or MD/PhD applicants as compared to MD applicants [8]. However, MD/MPH students had a higher rate of choosing primary care and had a higher number of interviews. Although MD/MPH students had significantly lower Step 1 and Step 2 scores as well as the number of ‘Honors’ grades in clerkships, it did not affect their percentage of matching as compared to MD students. This may indicate the strength of the dual degree in compensating for other relatively weaker areas in the application. Of note, MD/MBA students pursued more competitive specialty choices without any decrease in their overall percentage of matching into a residency program. The authors acknowledge that the Texas STAR database is voluntary and subject to survey and selection bias.

### Institutional Experience

We have been following the rise of dual degree programs over the past several years and wanted to explore the size, diversity of options, and underlying reasons for offering dual degree programs at other medical schools nationally. Our own experience with the dual degree programs has been very positive. Of all the dual degree programs offered in our school, only our MD/MPH, MD/PhD and MD/MBA student cohorts are determined pre-matriculation, as these are the largest programs and require independent coursework tailored to their unique schedules. Integrating the content for both degrees within four years has been logistically feasible because coursework for pre-matriculation degrees can begin in the summer prior to starting medical school. In addition, each degree program is allocated one half day per week in the pre-clerkship phase for coursework, with the remaining coursework completed during summer breaks and in the final phase of medical school. Masters degree programs that have capstone requirements can be completed longitudinally. Coursework with overlapping objectives to the MD degree have been approved by our university as shared credits between both degree programs and waivers exist for certain entrance exams such as the Graduate Management Admission Test (GMAT). Importantly, tuition for the master’s degree program is reduced for dual degree students. Thus far, we have encountered few problems related to academic promotion or drop out of students unable to fulfill Master's degree requirements. Integrating Masters content with the medical curriculum allows students to make and reinforce links to the content being learned as part of their medical degree. If students do not choose to pursue a dual degree, pathways of emphasis (no additional tuition) or certificate programs (reduced tuition compared to Masters programs) are attractive alternatives for students that can also provide more expertise and scholarly opportunities in a particular area of interest [26]. A national consortium of medical schools that offer dual degree programs would be helpful to establish a database and collect national data regarding cost and outcomes.

This study has several limitations. We chose to only include those institutions that average 10 or more dual degree master's program graduates per year and not percentages as we felt this was a more reliable method of identifying programs that had experience with consistent production of dual-degree graduates. Schools with a smaller cadre of graduating students may have different philosophies and approaches for developing these programs. There may be significant logistical barriers or lack of resources for these schools that should be considered. There is a significant investment of time and financial support needed to establish these programs for both the medical school and the school supporting the second degree, and some medical schools may have found that the number of interested applicants in their pool to be too small to justify the effort. In addition, several of the participants indicated that the demographic data provided by the AAMC and online listing of dual degree programs offered at their institution was not accurate. This could have impacted the sample of participating institutions in this study. We encourage schools to consider maintaining their own database of dual degree students and consider tracking residency career choice and career outcomes. The limitation of the lack of a single, comprehensive database has been noted by other authors [22].

## Conclusion

This report focused on evaluating the reasons that medical schools offer dual degree programs and how these support a growing recognition of a transdisciplinary approach to preparing medical students for a more complex, and rapidly evolving career in medicine. While this addresses a need for more broadly-based skills, it may lead to unintended consequences. Future studies should investigate the demographic differences of students who choose these programs, the impact of financial debt on students pursuing these programs, any difficulty in academic progression and promotion within dual degree students given the rigor required to complete an additional degree during medical school, and specialty choice. Follow up studies of alumni of dual degree programs are also needed to assess long-term impact on completion of residency training and choice of career setting.

Given the benefits described by respondents from our study, medical schools without dual degree programs should consider exploring the development of these programs or integrating these new disciplines into existing curricula.

### Supplementary Information

Supplementary Material 1.

## Data Availability

The data that support the findings of this study are available from the Association of American Medical Colleges (AAMC) but restrictions apply to the availability of these data, which were used under license for the current study, and so are not publicly available. Data are however available from the authors upon reasonable request and with permission of the AAMC.
