# Supplementary material for: Beyond the M.D.: Transdisciplinary approaches of high-volume dual degree M.D./Masters programs at U.S. allopathic medical schools
Source: BMC Med Educ. 2024 Jul 16;24:766. doi: 10.1186/s12909-024-05709-3 (PMC11253467; doi:10.1186/s12909-024-05709-3)
Supplement: Supplementary file 1 — Supplementary Material 1. [file 12909_2024_5709_MOESM1_ESM.docx]

**Appendix 1**

**Name:**

**Title:**

**Please confirm the AAMC data for your medical school for each of the following academic years:** 2019-2020, 2018-2019, 2017-2018, 2016-2017, 2015-2016, 2014-2015

Per Class Enrollment: (Number)

Programs offered:

MD/MS, MD/JD, MD/MPH, MD/MPhD, BS/MD, MD/MA, MD/MBA

Dual Degrees Granted in 4 Years:

Total Dual Degrees Granted:

**Demographics**

- Graduates Total:
- Graduates Male:
- Graduates Female:
- Black or African American Graduates:
- White Graduates:
- Asian Graduates:
- Hispanic, Latino, or of Spanish Origin Graduates:
- American Indian or Alaska Native Graduates:
- Native Hawaiian or Other Pacific Islander Graduates:
- Multiple Race/Ethnicity Graduates:
- Race/Ethnicity Unknown:
- Non-U.S. Citizen and Non-Permanent Resident Graduates:

Other Graduates:

**MD Tuition**

In State Tuition:

In State Tuition Health Insurance Fees:

Out of State Tuition:

Out of State Tuition Health Insurance Fees:

**Additional Dual Degree Program Information:**

Please indicate the reason(s) why your school chose to offer Dual Degree programs? •

Please indicate the reasons why your school chose to offer a dual degree BS MD programs?

Please indicate the reasons why your school chose to offer a dual degree MD MPH program?

Please indicate the reasons why your school chose to offer a dual degree MD MBA program?

Please indicate the reasons why your school chose to offer a dual degree MD PhD program?

Please indicate the reasons why your school chose to offer a dual degree MD JD program?

Please indicate the reasons why your school chose to offer a dual degree MD MS programs?

Please indicate the reasons why your school chose to offer a dual degree MD MA programs?

Which of your institution’s dual degree programs require students to commit pre-matriculation:

Has your school tracked differences in outcomes? If yes, please indicate differences in outcomes.

Has your school tracked outcomes of graduates of any of your dual degree programs? If yes, please indicate outcomes that have been tracked.

Are you aware of significant differences in student debt between MD only students and students in dual degree programs at your school? If yes, please describe your experience with this issue of debt.

Is debt a deterrent for students at your school in pursuing dual degree programs? How is debt a deterrent for students at your school in pursuing dual degree programs?

Are you aware of demographic differences between MD only and dual degree candidates? If yes, please describe the difference in demographics.

Do you have current plans to expand to more degree programs? If yes, please indicate which degrees you are considering.
